# Successful dabrafenib and trametinib combination therapy in a patient with recurrent *BRAF* ^V600E^ ‐mutant non‐small‐cell lung cancer and coexisting radiation pneumonitis

**DOI:** 10.1002/rcr2.1277

**Published:** 2024-01-24

**Authors:** Eriko Nakamura, Masahide Ota, Ryosuke Matsuda, Maiko Takeda, Tomomi Fujii, Yoshifumi Yamamoto, Shigeto Hontsu, Motoo Yamauchi, Masanori Yoshikawa, Shigeo Muro

**Affiliations:** ^1^ Department of Respiratory Medicine Nara Medical University Kashihara Japan; ^2^ Department of Cancer Genomics and Medical Oncology Nara Medical University Kashihara Japan; ^3^ Department of Neurosurgery Nara Medical University Kashihara Japan; ^4^ Department of Diagnostic Pathology Nara Medical University Kashihara Japan; ^5^ Department of Clinical and Investigative Medicine Faculty of Nursing Nara Medical University Kashihara Japan

**Keywords:** *BRAF*, dabrafenib, non‐small‐cell lung cancer, radiation pneumonitis, trametinib

## Abstract

There have been several reports of drug‐induced lung injury caused by molecular‐targeted agents. Additionally, medical history of interstitial lung disease and chest irradiation are established risk factors for the development and progression of drug‐induced lung injury. Moreover, the presence of fibrosis on chest computed tomography before treatment is a predictive factor for the appearance of pneumonia induced by anticancer drugs. Accordingly, patients with a history of interstitial lung disease or pneumonitis were excluded from clinical trials of dabrafenib and trametinib combination therapy for patients with previously treated *BRAF* ^V600E^‐mutant metastatic non‐small‐cell lung cancer. This article presents a case of successful dabrafenib and trametinib combination therapy in a patient with *BRAF* ^V600E^‐mutant non‐small‐cell lung cancer who had a history of radiation pneumonitis and developed recurrence after conventional chemoradiotherapy.

## Graphical abstract

Drug‐induced lung injury caused by molecular‐targeted agents has been reported. Medical history of interstitial lung disease and chest irradiation are established risk factors for the development and progression of drug‐induced lung injury. A case of successful dabrafenib and trametinib combination therapy in a patient with *BRAF* ^V600E^‐mutant non‐small‐cell lung cancer who had a history of radiation pneumonitis and developed recurrence after conventional chemoradiotherapy is presented in this article.

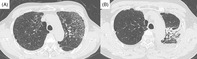

## INTRODUCTION

A medical history of interstitial lung disease and chest irradiation are established risk factors for the development and progression of drug‐induced lung injury.^1^ Additionally, the presence of fibrosis on chest computed tomography (CT) before treatment is a predictive factor for pneumonia caused by anticancer drugs.^2^ There have been several reports of drug‐induced lung injury caused by molecular‐targeted agents.^1^ Accordingly, patients with a history of interstitial lung disease or pneumonitis were excluded from an open‐label, phase two, trial of dabrafenib and trametinib combination therapy for patients with previously treated *BRAF* ^V600E^‐mutant metastatic non‐small‐cell lung cancer.^3^ This report describes the first reported case of successful combination therapy in a patient with recurrent *BRAF* ^V600E^‐mutant non‐small‐cell lung cancer who had coexisting radiation pneumonitis.

## CASE REPORT

**FIGURE 1:**
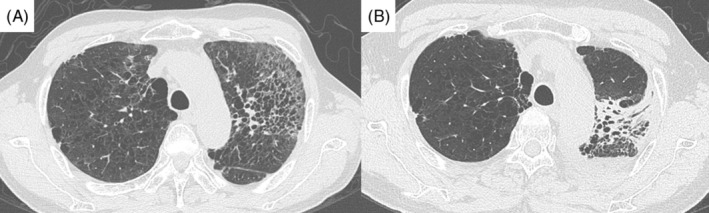
(A) Chest computed tomography scan obtained at the onset of radiation pneumonitis in April 2020. (B) A chest computed tomography scan was obtained when dabrafenib and trametinib combination therapy was initiated in August 2020.

**FIGURE 2:**
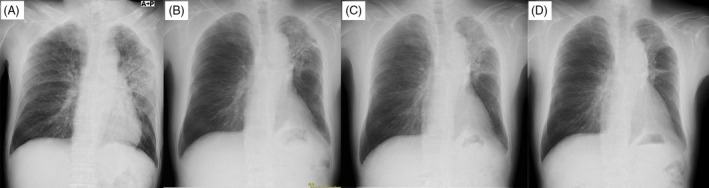
(A) Chest radiography scan obtained at the onset of radiation pneumonitis in April 2020. (B) Chest radiography scan obtained in July 2020. Radiation pneumonitis was improved by prednisolone. (C) Chest radiography scan obtained when the patient was started on dabrafenib and trametinib combination therapy in August 2020. (D) Chest radiography scan obtained in May 2021. As of 35 weeks after initiation of dabrafenib and trametinib combination therapy, the lung tumour shrinkage was maintained without worsening of interstitial pneumonitis.

A 60‐year‐old man with a smoking history presented to our department in November 2019 with a tumour in the left upper lung lobe and a swollen lymph node in the left hilar region. Bronchoscopy and cytology findings established a diagnosis of non‐small cell lung cancer; however, we could not collect sufficient tissue specimen for histology examination. Accordingly, chemoradiotherapy (CRT) for left upper lobe non‐small cell lung cancer (cT3N3M0, Stage IIIC) was started in December 2019. The CRT involved radiotherapy and concomitant weekly administration of nab‐paclitaxel and carboplatin. A CT scan obtained on February 2020 revealed lung tumour shrinkage; however, the patient presented symptomatic epilepsy due to metastatic brain tumours in March 2020. Accordingly, the patient underwent brain tumour resection, with genomic testing of the pathology sample revealing a *BRAF* ^V600E^mutation. After surgery, the patient underwent stereotactic radiation therapy (SRT) for the brain metastasis. Subsequently, the patient developed radiation pneumonitis and was started on prednisolone (PSL) at 30 mg/day in April 2020, which was subsequently tapered off (Figures 1A and 2A,B). Though we thought to initiate BRAF inhibitors after SRT, we did not initiate BRAF inhibitors because of radiation pneumonitis at that time. A CT scan performed in July 2020 revealed maintenance of the lung tumour shrinkage; however, contrast‐enhanced magnetic resonance imaging revealed new brain metastases.

In August 2020, the patient was admitted to our department for lung cancer treatment. The patient did not have a remarkable medical history or allergies. On admission, physical examination revealed the following vital signs: temperature, 35.9°C; blood pressure, 122/74 mmHg; pulse rate, 82 beats/min with regular cardiac rhythm; and percutaneous O_2_ saturation, 95% (on room air). Chest auscultation revealed intermittent inspiratory crackles in the left upper lung field.

Additionally, chest radiography revealed consolidation mixed with a large glass shadow in the left upper lung field (Figures 1B and 2C). Chest CT showed an 18 mm nodule shadow in the left upper lung lobe that had maintained its post‐CRT shrinkage. Additionally, there were fibrosis and increased contractile changes from the left upper lung lobe to the superior segment of the lower lobe. There were no findings suggestive of metastasis to other organs within the imaging range.

Blood tests did not reveal obvious inflammatory findings; moreover, Krebs von den Lungen 6 and surfactant protein D levels were within the normal ranges.

Following SRT for brain metastasis, the patient underwent oral combination therapy with dabrafenib (150 mg twice daily) and trametinib (2 mg once daily) for *BRAF* ^V600E^‐mutant metastatic non‐small‐cell lung cancer; additionally, the PSL dose (10 mg/day) for radiation pneumonitis was not altered. We considered tapering PSL down from 10 mg, but due to concerns about the appearance of drug induced pneumonitis and recurrence of radiation pneumonitis, the dose was cautiously considered for reduction. Twelve weeks after dabrafenib plus trametinib was initiated, the dose was reduced to 8 mg due to complaints of moon‐face appearance. Thereafter, after the PSL was reduced to 8 mg, a mild skin rash and cough without significant pneumonitis appeared. Thus, the PSL was continued at 8 mg, and the dose was not reduced thereafter due to concerns about worsening symptoms caused by PSL reduction. As of 35 weeks after initiation of combination therapy with dabrafenib and trametinib, the lung tumour shrinkage was maintained without worsening interstitial pneumonitis or recurrence of brain metastasis (Figure 2D).

## DISCUSSION

Mitogen‐activated protein kinase inhibitors, such as trametinib, have been shown to cause pneumonitis induced by targeted molecular therapy.^4^ Among targeted therapies for non‐small cell lung cancer, pneumonitis has been reported in 5% of patients with gefitinib, 4.5% with erlotinib, 0.7% with ALK inhibitors, such as crizotinib, ceritinib, and 6.5% with osimertinib therapy.^1^, ^5^ It has been also reported that 1.1% of patients using dabrafenib plus trametinib developed pneumonitis.^3^, ^6^ Although the risk of pneumonitis is not higher with dabrafenib plus trametinib than with other drugs, pneumonitis is sometimes fatal, warranting caution. In the present case, the patient had already developed radiation pneumonitis, which increased the risk of drug‐induced pneumonitis caused by the combination therapy. Therefore, we carefully monitored the interstitial shadows during combination therapy to check for worsening. Fortunately, we did not observe any marked deterioration. Since the patient had *BRAF* ^V600E^‐mutation‐positive lung cancer, there were concerns that the radiation pneumonitis might worsen and impede the administration of dabrafenib and trametinib combination therapy. PSL was administered to treat radiation pneumonitis since the pneumonia was predicted to worsen due to extensive irradiation of both the lung tumour and normal lung fields as well as due to atelectasis and G3 radiation pneumonia requiring oxygen administration. During follow‐up without treatment for lung cancer after the development of radiation pneumonitis, we only observed exacerbation of the brain metastasis; accordingly, brain tumour resection and SRT were performed to remove the brain metastasis. There was no subsequent worsening of the radiation pneumonitis; moreover, CT did not reveal significant ground‐glass shadows. This indicated that the radiation pneumonitis had stabilized. For the treatment of malignant melanoma, BRAF inhibitors can induce radiation sensitization, with reports of radiation recall dermatitis often occurring when BRAF inhibitors are started after radiation therapy.^7^ Radiation sensitization during radiotherapy and BRAF inhibitor combination therapy is not limited to the skin; radiation pneumonitis has also been reported. Such side effects have been reported with sequential as well as concurrent radiotherapy. Given that the combination of radiotherapy and BRAF inhibitors for brain metastases of melanoma may increase cutaneous radiation‐induced reactions,^7^ and the exclusion from a clinical trial of patients who had been treated with radiotherapy within 14 days,^3^ BRAF inhibitors were started at least 2 weeks after SRT. Trametinib has been reported to cause interstitial lung disease in real‐world clinical practice,^4^ accordingly, dabrafenib and trametinib combination therapy was initiated under PSL therapy, which did not worsen the interstitial pneumonitis. When the PSL dose was reduced to 8 mg, there was a worsening of symptoms. However, it was challenging to distinguish whether this deterioration was attributable to RP or was drug‐related. Consequently, the PSL dose was carefully maintained, considering the possibility that it was drug‐related. However, if the course of radiation pneumonitis remains stable, we believe that a reduction in the PSL dose should be contemplated to mitigate the side effects. However, since the severity of radiation pneumonitis varies across cases, it is necessary to make careful considerations regarding the safety of administering dabrafenib and trametinib combination therapy on a case‐to‐case basis, with close monitoring during treatment.

## AUTHOR CONTRIBUTIONS

Eriko Nakamura wrote the first draft of this manuscript. Masahide Ota, Ryosuke Matsuda, Maiko Takeda, Tomomi Fujii, Yoshifumi Yamamoto, Shigeto Hontsu, Motoo Yamauchi, Masanori Yoshikawa, and Shigeo Muro revised the article for intellectual content. All authors approved the final version of the manuscript.

## CONFLICT OF INTEREST STATEMENT

None declared.

## ETHICS STATEMENT

The authors declare that appropriate written informed consent was obtained for the publication of this manuscript and the accompanying images.

## Data Availability

Data sharing is not applicable to this article as no new data were created or analyzed in this study.
